# COVID-19 complicated by infective endocarditis or concomitant infection? a case report

**DOI:** 10.11604/pamj.2022.41.263.29438

**Published:** 2022-03-31

**Authors:** Chaymae Miri, Falmata Laouan Brem, Hammam Rasras, Noha El Ouafi, Bazid Zakaria

**Affiliations:** 1 Department of Cardiology, Mohammed VI University Hospital, Faculty of Medicine and Pharmacy of Oujda, Mohammed First University, Oujda, Maroc,; 2 Laboratory of Epidemiological of Clinical Research and Public Health, Faculty of Medicine and Pharmacy of Oujda, Mohammed First University, Oujda, Maroc

**Keywords:** COVID-19, infective endocarditis, complication, SARS-CoV-2, case report

## Abstract

Coronavirus disease 2019 (COVID-19) is a global pandemic and public health emergency. With a high infectivity and dissemination rate, cardiovascular complications have been observed and associated with a poorer prognosis. COVID-19 appears to be both a risk and prognosis factor for infective endocarditis. In this report, we present the case of a 53-year-old woman with a non-productive cough, progressive dyspnea and fatigue, diagnosed with COVID-19 four weeks earlier. The patient was referred to our department displaying the same symptoms. She was diagnosed with infective endocarditis of the mitral valve based on clinical symptoms, as well as radiological and analytical investigations. The patient was given appropriate medical treatment before admission based on azithromycin, corticosteroids for two weeks, during the hospitalization, she underwent treatment with antibiotics based on Teicoplanin and gentamicin. Outcome was good; the disappearance of the vegetative lesion on the weekly transthoracic echocardiogram (TTE). This rare case highlights questions about considering other coexisting diagnoses as well as possible complications a long with COVID-19.

## Introduction

The World Health Organization declared Coronavirus Disease 2019 (COVID-19) a global pandemic in March 2020 [1]. The severe acute respiratory syndrome coronavirus 2 (SARS-CoV 2) causes this illness, which causes significant morbidity and mortality and severe complications that endanger public health globally, and in particular cardiovascular complications are among the most important and potentially fatal [2, 3]. Besides, several diseases can complicate or coexist with SARS-CoV-2. We describe a rare case of mitral valve infectious endocarditis (IE) in a patient diagnosed four weeks earlier with COVID-19 infection. Two possible hypotheses were discussed; either COVID-19 complicated by infectious endocarditis because it can be a potential risk factor for IE or the patient had simultaneous COVID-19 infection and IE because the COVID-19 can have similar clinical features to those of IE. Currently, there is only a little data on IE associated or secondary to COVID-19.

## Patient and observation

**Patient information**: a 53-year-old female. She presents a non-productive cough, progressive dyspnea and fatigue, with a previous history of hypertension and an end-stage of kidney disease due to hypertensive nephropathy under hemodialysis through an arteriovenous fistula. The patient did not have a cardiac disease before and had not benefited from a cardiac ultrasound.

**Clinical findings**: on admission, we noted a respiratory rate of 20 breaths per minute, oxygen saturation of 88% in ambient air, heart rate at 94 beats per minute, blood pressure of 170/94 mmHg and temperature of 37,3°C. With turgidity of the jugular veins, hepato-jugular reflux. Cardiac auscultation revealed a mitral systolic murmur. Pulmonary auscultation revealed a bilateral fluid effusion syndrome.

**Timeline of current episode**: the patient presents a non-productive cough, progressive dyspnea and fatigue, RT-PCR (reverse transcription-polymerase chain reaction) was performed, returning positive for SARS-CoV-2, she was treated with azithromycin, corticosteroids, zinc and vitamin C. Four weeks later, the patient kept the same symptoms, and then she was referred to our hospital center.

**Figure 1:**
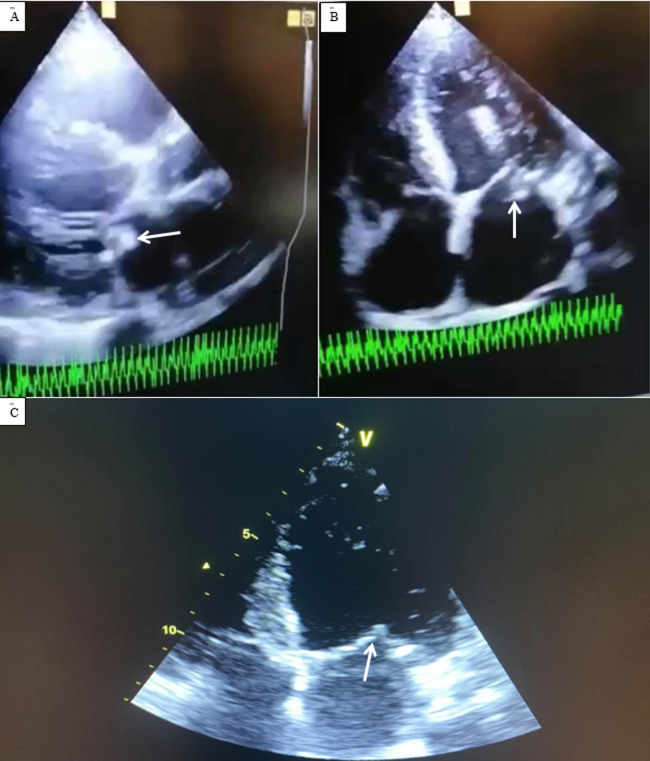
transthoracic echocardiogram: A) showing vegetation at the expense of the posterior mitral valve (white arrow); B) the four-cavity cut confirming vegetation at the level of the posterior mitral valve (white arrow); C) shows the disappearance of vegetation

**Figure 2:**
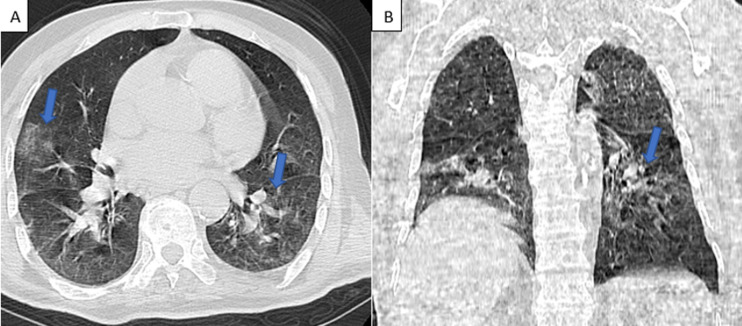
thoracic CT scan in the axial: A): and coronal plane; B): revealed 30% of CT abnormalities related to the COVID-19 infection

**Diagnostic assessment**: the electrocardiography showed regular sinus rhythm and left ventricular hypertrophy. Transthoracic echocardiography (TTE) showed vegetation on the posterior mitral valve prolapse in the OG, measuring 12*8mm (Figure 1A, B) Chest, abdomen and pelvis computed tomography revealed bilateral ground-glass pulmonary involvement, a focus of condensation and a defect in opacification of the right hypogastric branch (Figure 2) Biological analysis has shown a high level of ferritin (2080ng/mL), lymphopenia (490 element/mm^3^), moderate elevation in C-reactive protein (37 mg/L), fibrinogen of (4.2g/L) and lactate dehydrogenase (LDH) of (240UI/l), the PCR test for COVID-19 was negative and the COVID-19 serology was positive (IgG). A series of blood cultures were performed, returning negative.

**Diagnosis**: mitral infective endocarditis in a patient with a history of COVID-19 infection.

**Therapeutic intervention**: during the hospitalization, she underwent treatment with intravenous antibiotics based on Teicoplanin 800mg each three days for 28 days, and gentamicin 320mg one a day for 15 days.

**Follow-up and outcomes:** a follow-up echocardiography after two weeks revealed the disappearance of the vegetative lesion (Figure 1C). Clinically, the dyspnea had regressed. She had no adverse effects from the medications. The patient was discharged from the hospital on the 30^th^ after day admission without complications.

**Patient perspective**: during her hospitalization and following treatment, the patient and his family were pleased with the care they received and were optimistic about her condition's outcome.

**Informed consent**: the patient was told about the case report, why her case was unusual, and why the authors wanted to publish it. She knowingly consented to the writers' use of her photographs in this case report.

## Discussion

Infective endocarditis is a severe and complex condition with a mortality rate greater than 30% despite excellent therapy, and its association with COVID 19 is possible either as co-infection with IE or as a potential risk factor of IE. So far, there is limited evidence and information on COVID-19 and infectious endocarditis. COVID-19 infection and the resulting systemic inflammation may be risk factors for IE, especially in patients with underlying diseases who are susceptible. Yet the growth of the vegetation process begins with transient bacteremia; platelet aggregation and fibrinogen cleavage are caused by thromboplastin, released by tissue factor from damaged endothelium which can be observed during this infection [4]. Most of all, an infective endocarditis succeeding a COVID-19 pneumopathy may have as a hypothesis a bacterial pulmonary superinfection as a portal of entry responsible for an infective endocarditis, with corticosteroids weakening immunity and predisposing to serious infections, who but a direct causal link is not certain. This agrees with the study of Azin Alizadehasl *et al*. reported a case of infectious endocarditis of the prosthetic mitral valve three weeks after COVID-19 infection [5]. Our case presented a defect of opacification of the branch of the right hypogastric artery secondary possibly to the migration of the vegetation or to COVID-19. Various studies have shown that it could have caused a disruption of the coagulation cascade and contribute to vascular thrombosis [6].

There is a scarcity of data in the literature in treating therapy in patients with COVID-19 and IE. There is some evidence to support the treatment of angiotensin receptor blocker (ARB) or Angiotensin-converting-enzyme inhibitors (ACE-I) regardless of the ACE2 protein theory [7]. We will continue to prescribe ARBs to our patient because of the positive effects of this drug in patients with hypertension, heart failure, valvular heart disease, and chronic kidney failure. As this pandemic has spread worldwide and it affects millions of people, the combination between COVID-19 infection and other diseases will be frequent. Amir *et al*. showed the case of a patient with the diagnosis of infectious endocarditis concomitant with COVID-19 [8]. Moreover, in our case, it is not clear if IE complicates COVID, or she simultaneously had COVID-19 infection and EI because both can have the same clinical characteristics, which may be responsible for the late diagnosis of IE, especially when antibiotics were used, delaying early identification until the infection was highly advanced. Early screening for multiple diagnoses is essential to avoid late diagnosis and prevent complications that can be fatal. COVID-19 also concealed cardiac symptomatology, especially when antibiotics were used, delaying early identification until the infection was highly advanced.

## Conclusion

The Infectious endocarditis and COVID-19 infection are serious pathologies, so association of both will further worsen the prognosis, requiring further studies to fully understand this association and its management. This rare case highlights questions about considering other coexisting diagnoses and possible complications of the COVID-19 disease.

## References

[1] World Health Organization (2020). WHO announces COVID-19 outbreak a PANDEMIC March 12^th^, 2020 Coronavirus disease (COVID-19) outbreak Geneva. World Health Organization.

[2] Huang C, Wang Y, Li X, Ren L, Zhao J, Hu Y (2020). Clinical features of patients infected with 2019 novel coronavirus in Wuhan, China. Lancet.

[3] Driggin E, Mahesh Madhavan V, Behnood Bikdeli, Taylor Chuich, Justin Laracy, Giuseppe Biondi-Zoccai (2020). Cardiovascular considerations for patients, health care workers, and health systems during the COVID-19 Pandemic. J Am Coll Cardiol.

[4] McCormick JK, Tripp TJ, Dunny GM, Schlievert PM (2002). Formation of vegetations during infective endocarditis excludes binding of bacterial-specific host antibodies to Enterococcus faecalis. J Infect Dis.

[5] Alizadehasl A, Salehi P, Roudbari S, Peighambari MM (2020). Infectious endocarditis of the prosthetic mitral valve after COVID-19 infection. Eur Heart J.

[6] Tang N, Li D, Wang X, Sun Z (2020). Abnormal coagulation parameters are associated with poor prognosis in patients with novel coronavirus pneumonia. J Thromb Haemost.

[7] Sriram K, Insel PA (2020). Risks of ACE Inhibitor and ARB Usage in COVID-19: evaluating the evidence. Clin Pharmacol Ther.

[8] Amir M, Djaharuddin I, Sudharsono A, Ramadany S (2020). COVID-19 concomitant with infective endocarditis: a case report and review of management. Int J Infect Dis.

